# Hybrid Multisite Silicon Neural Probe with Integrated Flexible Connector for Interchangeable Packaging

**DOI:** 10.3390/s21082605

**Published:** 2021-04-08

**Authors:** Ashley Novais, Carlos Calaza, José Fernandes, Helder Fonseca, Patricia Monteiro, João Gaspar, Luis Jacinto

**Affiliations:** 1 International Iberian Nanotechnology Laboratory (INL), 4715-330 Braga, Portugal; carlos.calaza@inl.int (C.C.); jose.fernandes@inl.int (J.F.); helder.fonseca@inl.int (H.F.); joao.gaspar@inl.int (J.G.); 2 Life and Health Sciences Research Institute (ICVS), School of Medicine, University of Minho, 4710-057 Braga, Portugal; patriciamonteiro@med.uminho.pt; 3 ICVS/3B’s—PT Government Associate Laboratory, 4710-057 Braga/Guimarães, Portugal

**Keywords:** neural probe, neuroMEMS, silicon and polyimide microfabrication, flexible interconnect cable, interchangeable packaging, sensorimotor cortex, in vivo electrophysiology

## Abstract

Multisite neural probes are a fundamental tool to study brain function. Hybrid silicon/polymer neural probes combine rigid silicon and flexible polymer parts into one single device and allow, for example, the precise integration of complex probe geometries, such as multishank designs, with flexible biocompatible cabling. Despite these advantages and benefiting from highly reproducible fabrication methods on both silicon and polymer substrates, they have not been widely available. This paper presents the development, fabrication, characterization, and in vivo electrophysiological assessment of a hybrid multisite multishank silicon probe with a monolithically integrated polyimide flexible interconnect cable. The fabrication process was optimized at wafer level, and several neural probes with 64 gold electrode sites equally distributed along 8 shanks with an integrated 8 µm thick highly flexible polyimide interconnect cable were produced. The monolithic integration of the polyimide cable in the same fabrication process removed the necessity of the postfabrication bonding of the cable to the probe. This is the highest electrode site density and thinnest flexible cable ever reported for a hybrid silicon/polymer probe. Additionally, to avoid the time-consuming bonding of the probe to definitive packaging, the flexible cable was designed to terminate in a connector pad that can mate with commercial zero-insertion force (ZIF) connectors for electronics interfacing. This allows great experimental flexibility because interchangeable packaging can be used according to experimental demands. High-density distributed in vivo electrophysiological recordings were obtained from the hybrid neural probes with low intrinsic noise and high signal-to-noise ratio (SNR).

## 1. Introduction

Advances in microengineering and electromechanical systems (MEMSs) technology and microfabrication methods and materials have enabled the development of integrated high-density silicon (Si)-based neural probes for neuroscience applications [1,2,3,4,5,6,7,8]. The superior adaptability and reproducibility of Si microfabrication processes has allowed the continuous refinement of probes’ geometry parameters, which, in turn, has led to improved surgical implantation procedures, increased mechanical stability, and higher signal to noise ratio (SNR) neural recordings. Hence, it is currently possible to monitor the simultaneous activity of dozens to hundreds of individual neurons in multiple sites with these probes, which has been contributing to our understanding of information processing and coding in the brain and the development of more effective brain–machine interfaces [9,10,11,12].

More recently, there has been a rising interest in flexible neural probes and interconnect cables because they can reduce the mechanical mismatch between probe and brain tissue, permit fully implanted biocompatible cabling, and facilitate integration with flexible electronics [13,14,15,16]. However, and despite these advantages, flexible probes can buckle during brain insertion and require cumbersome mechanical rigidity augmentation strategies to increase the probe’s buckling force threshold during insertion/implantation [14,17,18]. Consequently, while probes fabricated on compliant substrates such as polyimide (PI), parylene C, or SU-8 can be seamlessly integrated with flexible interconnect cabling, they typically have limited geometry options. In particular, polymer probes with multishank designs for large-scale distributed neuronal recordings require complex augmentation strategies and have rarely been pursued [17,19,20,21,22]. Additionally, the polymer layers of integrated all-polymer probes are usually thicker than those used for cabling only because they must structurally support the implantable portion of the probe, thus leading to thicker and less flexible cabling.

Hybrid silicon/polymer neural probes that integrate microfabrication processes on both silicon and polymer substrates can be an alternative solution, given that different portions of the probe can be fabricated with different materials maximizing their potential benefits. This approach allows, for example, the combination of Si shafts with complex geometries, such as multisite/multishank designs that do not require additional brain insertion aids to increase the buckling force threshold, with thin fully flexible cabling for biocompatible electronics interfacing. Although these probes have received limited attention, previous examples have included Si probes with integrated flexible parylene C [23,24] or PI [25,26,27] cabling or alternating silicon/parylene regions [28]. In these examples, with the exception of [24], the silicon and polymer portions of the probe were fabricated separately and required postfabrication processes for bonding the cable to the probe. However, combining silicon and polymer micromachining into a single fabrication process, besides reducing fabrication complexity and costs, allows a higher degree of potential customization.

Nevertheless, even when flexible cabling is integrated, either in silicon or polymer substrates, there is still the need to perform an additional time-consuming packaging step of wire- or flip-chip bonding of the cable interconnect pads to a printed circuit board (PCB) used for electronics interfacing. Considering that different experimental demands and applications may require different probes, there is also the need for specific PCBs and electronics interfaces for each different probe. The inexistence of a standard for probe interfacing, while allowing some flexibility, also means that labs must invest in different types of connectors and packaging options for each probe and experiment, typically dictated by non-experimental requirements. Although fabrication techniques have been evolving toward more cost-effective, high-yield approaches, the use of Si probes is still forbidding for many neuroscience labs, especially for chronic experiments, with the cost of different packaging options adding up. This has limited the use and dissemination of probes, especially of those developed in labs [29]. The use of zero-insertion force (ZIF) connectors for electronics interfacing is an appealing solution to avoid definitive bonding to a PCB and is especially suited for flexible polymer cabling connections. ZIF connectors have been previously used not only in all-polymer neural probes [22,30,31] but also in Si neural probes with flexible cabling [26,32], albeit less frequently. These probes, however, still required the cumbersome postfabrication bonding process to connect the cabling to the probe as described above.

To facilitate wider dissemination and use, we propose here a new fabrication process for hybrid multisite silicon probes with monolithically integrated polyimide flexible interconnect cabling that allows interchangeable packaging options. By combining optimized fabrication processes for Si and PI, we fabricated several small-footprint multishank probes with a higher channel count (64 electrode sites) and thinner flexible polymer interconnect cabling (8 µm thick) than previously reported hybrid silicon/polymer probes [23,24,25,28,33,34]. By designing an integrated open-ended flexible connector pad that can mate with commercial zero-insertion force (ZIF) connectors on the PCB side, definitive packaging for these probes is avoided allowing great experimental flexibility. With this design, the same probe can be easily connected to different custom-designed PCBs for each required application, or different probes, with different geometries and layouts, can use the same interface PCB. Additionally, by removing the definitive bonding of the probe to the connector, the speed of fabrication increases while overall costs decrease. This also leads to improved wafer utilization, which, when combined with optical lithography near its patterning limits, as we show here, can increase the number of probes fabricated per wafer, further lowering costs.

**Figure 1:**
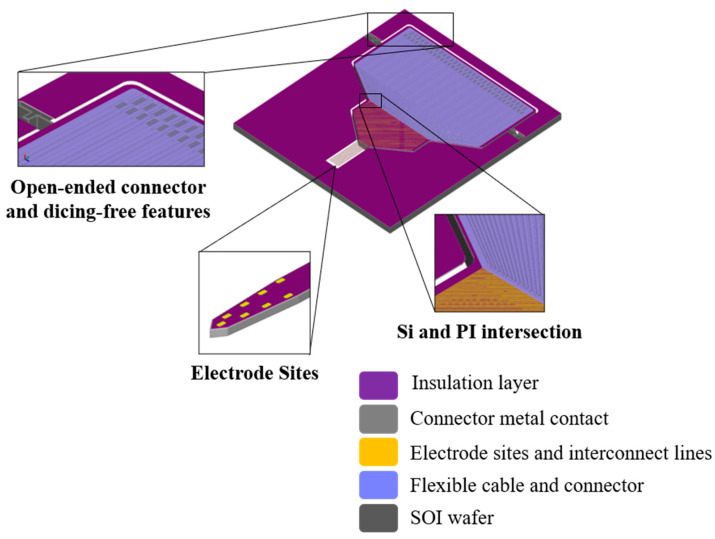
Three-dimensional (3D) schematic of the neural probe.

Figure 1 shows a schematic of our hybrid multisite silicon neural probe with integrated flexible polyimide cable. This paper describes the design, fabrication, and in vivo electrophysiological assessment of these probes.

## 2. Materials and Methods

### 2.1. Microfabrication of the Neural Probe

**Figure 2:**
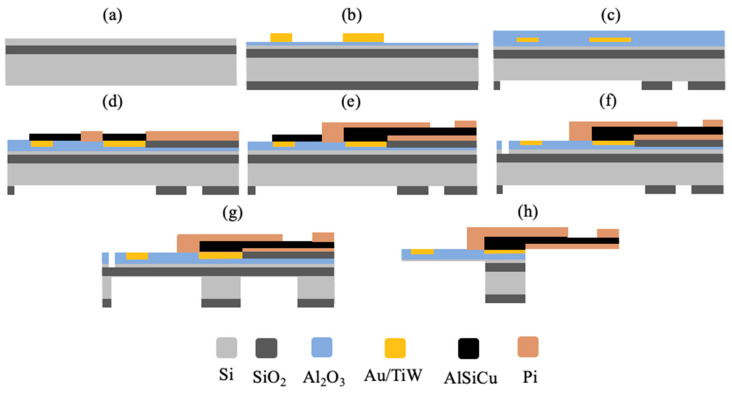
Neural probe main fabrication steps. (**a**) Silicon-on-insulator (SOI) wafer; (**b**) Au/TiW thin-film patterning and back-side SiO_2_ layer deposition; (**c**) font-side Al_2_O_3_ passivation and back-side SiO_2_ layer patterning; (**d**) electrode site protection and SiO_2_ and PI patterning; (**e**) interconnector AlSiCu and PI patterning; (**f**) front-side Si DRIE; (**g**) back-side Si DRIE; (**h**) hydrogen fluoride (HF) SiO_2_ release. NB: not to scale.

The neural probe was fabricated using standard semiconductor micromachining processes. Briefly, 200 mm silicon-on-insulator (SOI) wafers (15 µm device layer, 2 µm buried oxide, 625 µm handle wafer) (SVM, Santa Clara, USA) were used as substrate (Figure 2a). The back side was protected with one extra micron of SiO_2_ deposited by plasma-enhanced chemical vapor deposition (PECVD) as a hard mask, and a layer of 500 nm Al_2_O_3_ was sputtered for front-side passivation. A metal stack layer of 15 nm TiW/150 nm Au/5 nm Cr was then sputtered and patterned via reactive ion etching (RIE) (Figure 2b). This was followed by deposition of a 500 nm layer of Al_2_O_3_ for passivation defined by wet etch, and the back side was patterned for DRIE (Figure 2c). To proceed with the definition of the polyimide (PI) connector, electrode sites were first protected with a stack of 500 nm AlSiCu defined via wet etch. A 500 nm SiO_2_ sacrificial layer for PI release was then patterned via RIE, followed by a 3.75 µm thick layer of PI (PI-2611, HD MicroSystems, Parlin, NJ, USA), which was spin-coated and cured at 250 °C for 14 h and etched via RIE to open vias (Figure 2d). Then, the AlSiCu metal stack (1000 nm AlSiCu/150 nm TiW/200 nm AlSiCu/50 nm TiW) of the interconnector was patterned via metal RIE, followed by the deposition of another 3.75 µm thick layer of PI, and connection pads were patterned via PI RIE for connector definition (Figure 2e). To define the probe shanks, the Al_2_O_3_ passivation layer was etched to allow Si DRIE on the front side. Al was then wet-etched to remove the protection of the device area, and Cr was wet-etched to expose the Au sites (Figure 2f). After 15 µm probe definition, the front side was protected using Cool Grease (AI Technology Inc., Princeton Juntcion, NJ, USA) for wafer bonding to a handling wafer. Si (625 µm) on the back side was etched via DRIE (Figure 2g), followed by hydrogen fluoride (HF) vapor etch to remove the buried oxide layer and release the PI connector (Figure 2h). Finally, the handling wafer was released in a water bath (60 °C) and cleaned with acetone. Because dicing the wafer reduces wafer yield, a dicing-free process [35] was implemented for individual probe release from the wafer. For that purpose, a 50 μm wide trench around the device was patterned with DRIE on the front and back sides. U-shaped breakout beams connect the side of the probes to the bulk wafer, allowing safe wafer handling during the process and effortless device release by simply breaking the beams with tweezers.

The fabrication process was also optimized at wafer level, which allows the scale-up of the fabrication of probes with different geometries and layouts within the same wafer.

### 2.2. Neural Probe Packaging

For signal acquisition for the impedance measurements and in vivo electrophysiological recordings, the probe was packaged with a custom-designed PCB with a flexible printed circuit (FPC) ZIF connector (FH39A-67S-0.3SHW, Hirose Electric, Tokyo, Japan) on the probe side and two Omnetics connectors (A79022-001, Omnetics Connector Corporation, Fridley, MN, USA) on the acquisition system side. Because commercial ZIF connectors require a minimum cable thickness to guarantee secure and electrical connection (0.3 mm in this case), a 0.29 mm thick polypropylene spacer was used inside the connector to guarantee that the 8 µm polyimide connector pad of the integrated flexible cable was appropriately secured and connected.

### 2.3. Electrical Impedance Analysis

Prior to electrode site impedance measurements, the tips of the neural probe shanks were immersed in a solution of 50 mM potassium hydroxide (KOH) and 25% hydrogen peroxide (H_2_O_2_) for 10 min, as described in [36], to remove fabrication process residues and obtain clean gold electrode sites. Shanks were then rinsed in abundant Milli-Q water. Electrode site impedance was measured with nanoZ (White Matter LLC, Seattle, WA, USA) in phosphate-buffered saline solution (PBS 1×) at 1 kHz.

### 2.4. In Vivo Electrophysiological Recordings and Analysis

Wild-type mice (n = 3) were anesthetized by an intraperitoneal injection mix of ketamine (75 mg/Kg) and medetomidine (1 mg/Kg) and positioned in a stereotaxic frame (World Precision Instruments). The surgical procedure consisted of exposing the skull following a midline skin incision and drilling a burr hole above the motor and somatosensory cortices (centered at −1.0 mm AP and 1.5 mm ML from bregma, according to [37]). A 5 cm long, 8-shank neural probe with 64 electrode sites connected to the custom-designed PCB via the FPC ZIF connector was attached to a micrometric stereotaxic arm (1760, Kopf Instruments, Los Angeles, CA, USA). The PCB was then connected to a headstage (RHD2132, Intan) for signal acquisition. Following dura removal, the probe was lowered into the brain, through the burr hole, to a depth of at least −0.5 mm (DV) from the brain surface. A stainless-steel screw, positioned in another burr hole at the back of the skull, was connected to the PCB ground pad. Spontaneous extracellular neuronal activity signals were acquired with an Open Ephys acquisition system [38] at 30 Ks/s.

Extracellular neuronal recordings were analyzed with custom-written MATLAB code (MathWorks), and initial spike sorting was performed using JRClust [39]. Recordings were filtered between 0.6 and 6 kHz, and spikes were detected using an amplitude threshold at least 5 times higher than the background noise standard deviation. The manual curation of single-unit clusters, after JRClust initial automatic spike sorting, was performed by visual inspection of interspike interval histograms, auto- and cross-correlograms, and clusters’ spike waveforms.

The signal-to-noise ratio (SNR) was calculated using the formula: SNR = $\frac{RMS\;spike}{RMS\;noise}$, where *RMS spike* is the average of the root mean square of 1 ms windows centered at the peak of each detected spike, and *RMS noise* is the average standard deviation of the portions of the extracellular signal where spikes did not occur.

### 2.5. Histology

To determine insertion tracks and neural probe positioning in the brain, probe shanks were coated in DiI stain (1,1′-dioctadecyl-3,3,3′,3′-tetramethylindocarbocyanine perchlorate) (D3911, Thermo Fisher, Waltham, MA, USA) prior to brain insertion. DiI is a lipophilic red fluorescent dye that has been used to determine neural probes’ positioning after implantation [11,40]. At the end of the recording session, animals were transcardially perfused with 0.9% saline followed by 4% paraformaldehyde (PFA) in PBS, and the brain was carefully removed and stored in 4% PFA overnight. Brains were then serially sectioned at 100 µm in a vibratome (VT 1000S, Leica, Wetzlar, Germany). Slices were counterstained with DAPI nucleic acid stain (1:1000) (A1001, PanReac AppliChem, Barcelona, Spain), mounted on coverslipped slides with mounting medium (Shandon Immu-Mount, 9990402, Thermo Fisher), and imaged in a confocal fluorescence microscope (FV3000, Olympus, Tokyo, Japan).

## 3. Results and Discussion

**Figure 3:**
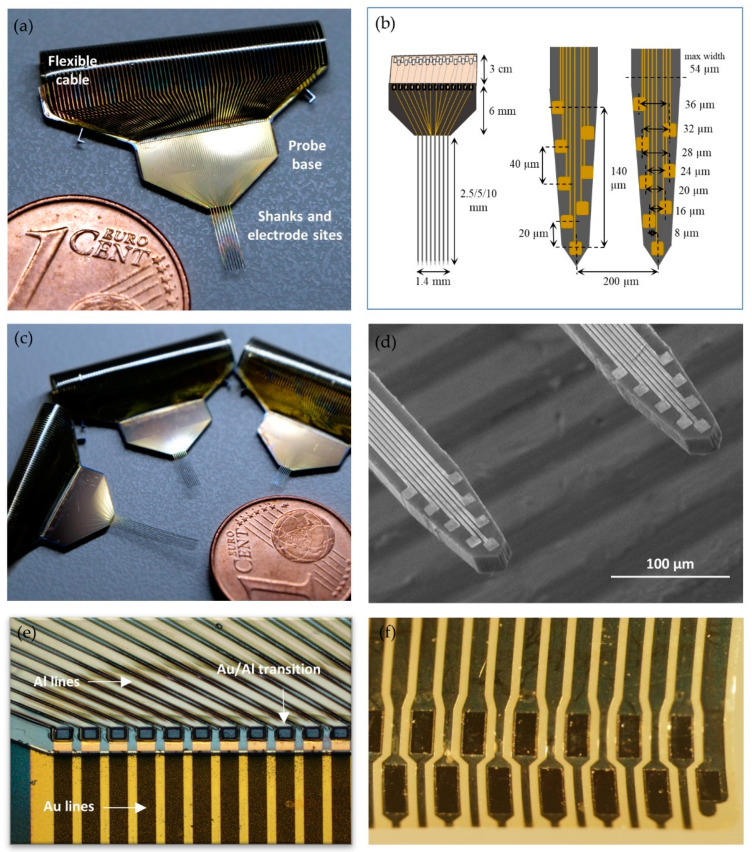
Fabricated neural probe: (**a**) photograph of a 5 mm long silicon probe with a polyimide flexible cable; (**b**) schematic drawing of a probe with respective dimensions showing a full probe view (**left**) and the detail of two shanks (**right**). NB: image not to scale; (**c**) three fabricated probes with three different shank lengths, 2.5, 5, and 10 mm; (**d**) SEM image of two shanks of a neural probe, with 8 gold electrode sites and respective interconnect lines in each shank; (**e**) monolithic integration of the flexible polyimide cable. Detail of the intersection zone of gold (Au) and aluminum alloy (Al) interconnect lines from the silicon and polyimide portions of the probe, respectively; (**f**) microphotograph showing detail of connector pad of the flexible polyimide cable.

Figure 3a shows an example of a fabricated neural probe, and Figure 3b displays a simplified schematic drawing of the probe with all respective dimensions. The probes were designed and fabricated with 8 shanks with a pitch of 200 μm and 8 electrode sites per shank, for a total of 64 electrode sites. Each shank has a maximum width of 54 μm and a thickness of 15 μm, and three different shank lengths were produced (2.5, 5, and 10 mm, Figure 3c). Gold electrode sites on shank tips have an area of 72 μm^2^ (6 μm × 12 μm) and are distributed vertically in two columns along the two edges of each shank with a vertical pitch of 40 μm (except for the two bottom electrode sites, which have a vertical pitch of 20 μm) (Figure 3d).

The employed monolithic fabrication process relied on optimized fabrication processes for Si and PI and can be described in two separate parts: fabrication of the Si multishank probe and fabrication and monolithic integration of the flexible PI interconnect cable.

### 3.1. Design and Fabrication of Multishank Silicon Probe

The substrate of choice for the Si neural probe was a silicon-on-insulator (SOI), which allowed precise control of the neural probes’ implantable shank thickness and that other parts of the probe remained at full wafer thickness [32]. The thickness of the shanks was determined by the SOI device layer, which was 15 µm, while the probe’s base remained at wafer thickness (approx. 640 µm) to provide support for safer handling. Other hybrid probes with integrated flexible interconnect cables that did not use SOI wafers reported considerably higher probe thicknesses (50–150 µm) [24,34], which can cause increased brain tissue damage and reactive responses upon insertion/implantation [41,42]. At 15 µm thick, the shanks on our probes are small enough to minimize tissue displacement and brain damage upon insertion and sufficiently rigid to avoid buckling and provide great mechanical stability.

Alumina (Al_2_O_3_) was chosen for neural probe passivation. Although SiO_2_ is most commonly used for this purpose, it was not compatible with the implemented fabrication process because it was used as the sacrificial layer for PI release. Additionally, alumina is biocompatible and chemically stable for chronic implantation [43]. Parylene or PI have also been previously used as insulators for other Si neural probes with flexible integrated interconnect cables [33,34], but they significantly increased the thickness of the implanted portion of those probes when compared with ours. Although using PI as a passivation layer could facilitate the monolithic integration of our PI cable, the use of a 500 nm thick alumina passivation layer ensured that probe thickness was kept to the minimum (which would have otherwise increased if a 3–7.5 µm thick polyimide passivation layer was used as in [24,28,44]). The undesirable increase in thickness would also defeat the purpose of using a SOI wafer for precise control of reduced shank thickness.

Three different shank lengths were fabricated: 2.5, 5, and 10 mm (Figure 3c). Although the fabrication process allowed the longest shank design (10 mm), a noticeable bend of the shanks with this design was observed (14.2 ± 1.10° angle), most likely due to thin-film stress, and was not used in further experiments. Longer shanks (>5–6 mm) in high-aspect-ratio silicon neural probes can bend if layers with different tensile and compressive forces are not properly balanced during the fabrication process [26,45,46]. This can, however, increase the fabrication complexity. The shorter fabricated shank lengths showed no or negligible bending (no bending for the 2.5 mm long shank; 3.9 ± 0.3° angle for the 5 mm long shank).

**Figure A1:**
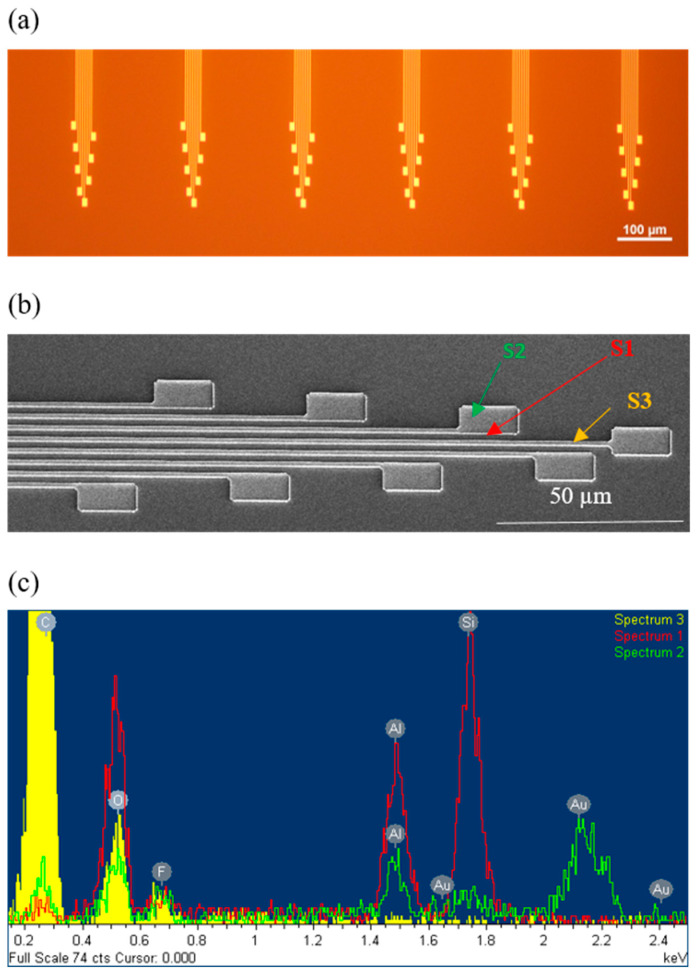
Structural characterization of electrode sites and interconnect lines: (**a**) Optical microscopic image of the electrode sites after metal patterning; (**b**) SEM image of the electrode sites after metal dry etch (S1: interline space; S2: center of electrode site; S3, wall of metal line); (**c**) EDX to confirm metal etch in between interconnect metal lines (peaks from S1: Si and Al_2_O_3_; S2:Au; S3:C).

Each shank on the neural probe has eight gold electrode sites distributed along the two edges of the shank (Figure 3d). This electrode site layout increases spatial sampling while also permitting the over-representation of neural activity across different nearby sites, which facilitates spike sorting and increases single-unit yield and separation [7,47]. Sputtered gold was the chosen metal for the electrode sites, but the same fabrication process could be employed using another metal with suitable electrical impedances. Gold sites have the advantages of being biocompatible and not requiring additional electrodeposition procedures to lower impedances to the desired range, at least with the site dimensions used here. Each gold interconnect metal line arising from each electrode site has a width of 2 μm, which equals the patterning resolution limit of the implemented low-cost lithography process. At 54 μm, shanks have the minimum possible width to accommodate all electrode sites and the interconnect metal lines. Energy-dispersive X-ray spectroscopy (EDX) structural analysis of the electrode sites and interconnect metal lines can be found in Appendix A (Figure A1).

### 3.2. Fabrication and Monolithic Integration of Flexible Polyimide Cable

To avoid time-consuming postfabrication bonding processes to connect the flexible cable to the silicon probe, such as in [26,32,48], a microfabrication process based on PI was used here to monolithically integrate a polyimide interconnect cable in the probe. The metal interconnect lines from the electrode sites travel up along the shanks and end in larger gold metal pads (100 × 100 μm) on the base of the Si probe. These larger pads form the transition zone where the electrodes’ gold interconnect lines contact with the flexible polyimide (PI) cable aluminum alloy intermetal lines (Figure 3e). PI was the chosen substrate for the flexible cable due to its conformational rigidity, dielectric properties, and process compatibility [49]. An aluminum alloy (AlSiCu) was used as an interconnector metal on the PI cable because it is not only more affordable than a noble metal but also displays good adhesion properties to Si and PI and low residual stress [50]. The area of the interconnect metal pads of the Si/PI transition zone, both on the probe and cable sides, as well as the width of the interconnect lines on the probe base, could be further reduced to create a smaller probe that would be more amenable for experiments requiring chronic brain implantation. The fabricated cable is only 3 cm long but could also be extended to any desired length without compromising the fabrication process or increasing the electrical resistance of the interconnect lines. Nevertheless, the 8 µm thick polyimide cable is the thinnest ever reported for a hybrid silicon/polymer neural probe, which could be beneficial for cabling bioimplantation.

**Figure A2:**
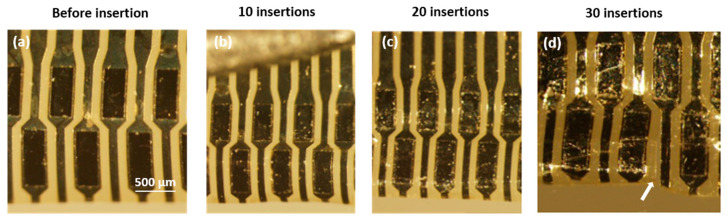
Structural characterization of flexible connector reinsertion cycles: (**a**) optical microscopic image of connector pads before any insertion (**a**) after 10 (**b**), 20 (**c**), and 30 (**d**) reinsertions. After 30 reinsertions, a tear is visible in the polyimide between the metal pads/interconnect lines (white arrow).

The flexible interconnect cable was also designed to terminate in a pad array with a custom format for effortless insertion into a commercial ZIF connector (Figure 3f). The connector package creates an interface between the electrode sites and the external electronics for signal acquisition. Si probes typically have rigid interconnects that require time-consuming wire- or flip-chip bonding processes to physically connect them to an interfacing PCB. Additionally, commercial probes also have a limited number of packaging options an experimenter can choose from. The approach presented here avoids definitive physical bonding of the probe to the electronics interface and allows interchangeable packaging according to the experimental/application demands. With this approach, the same neural probe can be easily connected to different custom-designed PCBs for each required application, or different probes, with different geometries and layouts, can use the same interface PCB. The use of ZIF connectors for neural probes has been previously shown to be an advantageous approach for experiments requiring experimental flexibility [22,26,30]. Although versatile, thin-film flexible connectors that undergo repeated cycles of connector insertion can fail due to mechanical damage, which can limit their long-term use [51]. Assessment of the structural and electrical properties of the flexible cable pad connector through repeated cycles of insertion into the FPC connector can be found in Appendix A (Figure A2). From our experience, the polyimide connector of the flexible cable can be easily connected to the FPC connector of the PCB up to 30–40 times without material or electrical failure. From that point on, although the electrical resistance of the connector pads continues to be very low, the connector tends to form tears in the polyimide between the metal pads/lines, which eventually leads to connector failure.

### 3.3. Electrical Impedance of Neural Probe

Following the connection of the probe to the custom PCB and electrode site gold surface cleaning, electrode electrical impedance was measured. The average electrode impedance measured at 1 kHz in phosphate-buffered saline (PBS) was 441.97 ± 47.98 kΩ. The measured mean electrical impedance of the electrode sites was within the optimal range for neural recordings with high SNR [52].

### 3.4. In Vivo Electrophysiology

**Figure 4:**
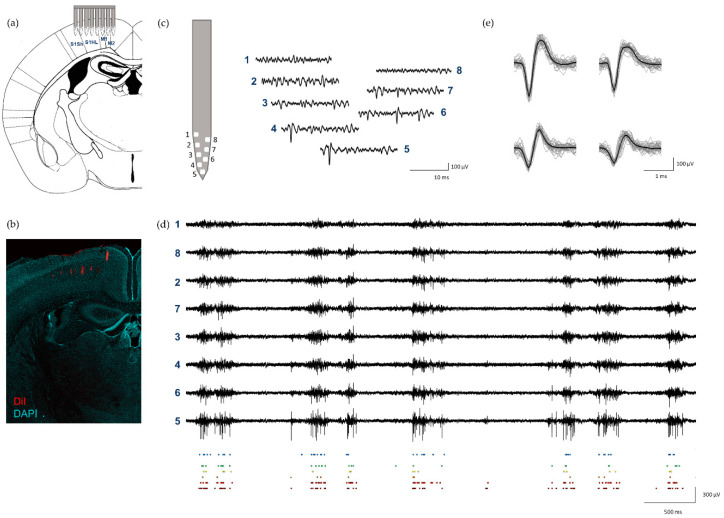
In vivo recordings in the mouse cortex: (**a**) schematic representation of probe insertion in motor and somatosensory cortices; (**b**) neural probe tracks (red) in the cortex; (**c**) example of neuronal activity (15 ms) simultaneously recorded from 8 electrode sites from shank 1 of the probe (signals band-pass filtered between 0.3 and 6 kHz); (**d**) example of longer signal traces (5 s) of neuronal activity recorded from 8 electrodes sites from shank 1 (filtered 0.3–6 kHz, black traces, top) and respective raster plot of detected spikes from each electrode site (colored squares, bottom); (**e**) four isolated single units from shank 1 from the same recording shown in (**d**), displaying mean waveform (black) and the first 100 spike waveforms (gray).

To assess the fabricated neural probes’ performance in the context of an in vivo experiment, brain electrophysiological extracellular recordings were performed, as depicted in Figure 4a. Neuronal activity from the motor and somatosensory cortices of anesthetized mice (Figure 4b) was recorded from probes with a high signal-to-noise ratio (SNR) across electrode sites and permitted subsequent reliable spike sorting. Figure 4c,d shows example traces of neuronal activity recorded from eight electrode sites in one shank of the probe. The mean RMS of the recorded extracellular signals across electrode sites was 5.29 ± 0.6 µV with a SNR_Voltage_ of approximately 7.1 ± 0.6. The recorded neural signal RMS was comparable to other state-of-the-art neural probes [3,4]. Due to the excellent SNR, it was possible to detect high-amplitude spikes (Figure 4d), which were then reliably isolated into several single-unit clusters from all shanks. Figure 4e shows the waveforms of four single units isolated from one shank of the probe during a 5 min recording.

## 4. Conclusions

A hybrid silicon neural probe with monolithically integrated polyimide flexible cabling and an open-ended connector for interchangeable packaging is presented. The monolithic fabrication process described permitted the integration of a multishank, multisite, 64-channel silicon (Si) neural probe with an 8 µm thick highly flexible polyimide (PI) cable. When compared with previously published hybrid neural probes with flexible cables, the one described here presents a significantly higher number of electrode sites and a thinner interconnect cable. Additionally, an open-ended connector pad was designed at the end of the flexible cable to permit the use of any desired printed circuit board (PCB) for probe interfacing, as long as a ZIF connector is present on the PCB side. This allows great experimental flexibility because the packaging can be easily changed to meet experimental demands without alterations to the probe or cable connector. High SNR (approx. 7) and low intrinsic noise (5.29 ± 0.6 µV) neuronal recordings were obtained in mice with this neural probe. Future work to deploy and assess the performance of the probe in a chronic setting will contribute to the wider dissemination of neural probes and the empowerment of the neuroscience community to use sophisticated neuroengineering tools.

## Data Availability

Data sharing is not applicable to this article.

## Appendix A

### Appendix A.1. Neural Probe Structural Characterization

SEM characterization was performed directly at the water level without any preceding sample preparation, thus avoiding the introduction of artifacts into the measurements. SEM was performed to structurally evaluate the fabrication of the probes with a NovaNanoSEM 650 (FEI) imaging tool coupled with an energy-dispersive X-ray spectroscopy (EDX) Inca (Oxford Instruments) detector.

To confirm if metal etch was successful between metal lines, after metal patterning, SEM with EDX was performed (Figure A1). Spectrum 1, taken from the interline space, shows that silicon (Si) and aluminum (Al) are the most prevalent elements corresponding to the wafer composition and respective passivation. Other elements present at this step of the process, such as carbon (C) and oxygen (O), correspond to the photoresist used as a mask for the metal dry etch and etching residues. Spectrum 2 was taken from the center of the electrode site and shows Au as the major element. Spectrum 3 was taken from a wall of a metal line, which contains dry etch residues and photoresist mainly composed of C and O (Figure A1b,c) that posteriorly would be covered with the passivation layer.

### Appendix A.2. Structural and Electrical Assessment of Flexible Connector Reinsertion Cycles

The effect of multiple reinsertions of the flexible connector into the FC/PC connector of the packaging PCB was assessed by repeating the insertion process 30 times. Optical microscopic photographs of the connector pads were taken every 10 insertions for the visual inspection of material deterioration/failure. Additionally, electric resistance measurements of the pads were also taken every 10 insertions with a multimeter.

The flexible polyimide cable allowed stable reinsertions up to 30 times, as evidenced by low and consistent resistance measurements and reduced deterioration of metal contact pads and/or polyimide. Figure A2 shows optical microscopic images of the connector pads taken before the first insertion and during successive reinsertion cycles. Although polyimide became increasingly crumpled and minor wear was detected on the metal pads as the number of reinsertions increased, mechanical failure was only detected after 30–40 insertions. After this number of reinsertions, the polyimide between the metal pads/lines started to tear (Figure A2d), which eventually led to further mechanical damage and increased difficulty in connecting the flexible connector pad to the FPC connector on the PCB. The electric resistance of the connector pads remained approximately the same during 20 insertions, with a measured increase of 50% resistance after 30 insertions. The obtained measurements were the following: 2.2 Ohm before insertion, 1.9 Ohm after 10 insertions, 2.2 Ohm after 20 insertions, and 3.5 Ohm after 30 insertions. Even with a 50% increase after 30 insertions, the measured resistance was very low.
